# Endoscopic-Assisted Linea Alba Reconstruction plus Mesh Augmentation for Treatment of Umbilical and/or Epigastric Hernias and Rectus Abdominis Diastasis – Early Results

**DOI:** 10.3389/fsurg.2016.00027

**Published:** 2016-05-13

**Authors:** Ferdinand Köckerling, Marinos Damianos Botsinis, Christine Rohde, Wolfgang Reinpold

**Affiliations:** ^1^Department of Surgery, Centre for Minimally Invasive Surgery, Vivantes Hospital Berlin, Academic Teaching Hospital of Charité Medical School, Berlin, Germany; ^2^Department of Surgery, Wilhelmsburger Hospital Groß Sand, Academic Teaching Hospital of University Hamburg, Hamburg, Germany

**Keywords:** rectus abdominis diastasis, umbilical hernia, epigastric hernia, linea alba reconstruction, video-endoscopic technique

## Abstract

**Introduction:**

Symptomatic umbilical and/or epigastric hernias are often seen concomitantly with rectus abdominis diastasis (RAD), and suture repair of such defects has a high recurrence rate. In the literature, there are reports of both endoscopic and open techniques for repair of symptomatic umbilical and/or epigastric hernias in association with RAD. This paper now reports on the early results of a hybrid technique used for reconstruction of the linea alba and mesh augmentation [endoscopic-assisted linea alba reconstruction plus mesh augmentation (ELAR plus)].

**Materials and methods:**

Between 15 June 2015 and 31 January 2016, 40 patients with symptomatic umbilical and/or epigastric hernia and concomitant RAD underwent reconstruction of the linea alba using a hybrid technique involving a small umbilical incision and the use of video-endoscopic equipment. The patients comprised 29 men and 11 women with a mean age of 53.6 years and mean BMI of 32.6. The mean operating time was 120 min. The mesh had a mean longitudinal extension of 18.6 cm and transverse extension of 9.1 cm.

**Results:**

Thirty-day follow-up results are available for all patients. Thirty-seven out of 40 patients (92.5%) experienced no postoperative complication. Two cases of discrete impaired umbilical wound healing and one seroma were successfully managed with conservative treatment. On 30-day follow-up, 3 out of 40 patients (7.5%) complained of intermittent pain on exertion, and 2 out of 40 patients (5%) still took painkillers when required.

**Conclusion:**

ELAR plus is a novel minimally invasive procedure for repair of symptomatic umbilical and/or epigastric hernias with concomitant RAD. Reconstruction of the linea alba *via* a minimally invasive access route is able to restore the normal anatomy of the abdominal wall.

## Introduction

Rectus abdominis diastasis (RAD) describes a condition in which the two rectus abdominis muscles are separated by an abnormally wide distance (1). Any separation of more than 2 cm is considered to be abnormal (1). RAD is usually quite apparent on physical examination (1). When a patient with RAD raises his or her head and begins to sit up, the increase in intra-abdominal pressure as the two rectus muscles contract can result in a diffuse fusiform bulge (1). The linea alba can become thinned due to stretching, which can be caused by elevated intra-abdominal pressure, such as in pregnancy and obesity (1). Patients with RAD typically have one of two profiles: middle-aged and older men with central obesity, or small, fit women who have carried a large fetus or twins to term (1).

It has been demonstrated that RAD produces deterioration in the functions of the abdominal wall with associated muscular imbalance and chronic back pain (2).

Rectus abdominis diastasis is often seen in association with primary abdominal wall hernia (umbilical and/or epigastric hernia) (3). For example, Köhler et al. (3) diagnosed RAD in 45% of patients with small (<2 cm) umbilical and epigastric hernias. The 31.2% of those patients with small umbilical and epigastric hernias, with concomitant RAD, who underwent suture repair had a significantly higher recurrence rate after mean follow-up of 31 months compared with non-RAD patients (3). The authors concluded that even small umbilical and epigastric hernias frequently occur concomitantly with RAD. The results support the theory that midline hernias, regardless of size, with concomitant rectus diastasis require mesh repair due to unacceptably higher recurrence rates (3).

At present, there is no consensus among the international surgical community on the surgical treatment of RAD as regards indications or surgical techniques (2). If RAD is symptomatic or is associated with midline hernias (umbilical and/or epigastric), corrective surgery of all pathologies at the same time could represent the most recommended approach (2).

There are many methods with which RAD can be repaired (1). These differ by approach (open versus laparoscopic), numbers of layers of sutures, the position of suture placement, suture material used, and whether or not mesh is used (4).

Laparoscopic plication of the linea alba is recommended as a mesh-free approach for treatment of RAD (5). Plication of the linea alba can be combined with mesh augmentation in the IPOM technique for enhanced stabilization of the abdominal wall (6).

Alternatively, following umbilical incision an endoscopic procedure can be performed in a space created between the subcutaneous tissue and the anterior layer of the rectus sheath (2), while repositioning or resecting the contents of an umbilical or epigastric hernia. Next, *via* the defects caused by rectus diastasis, meshes are introduced into the preperitoneal space where they are fixed. This is followed by endoscopic plication of the anterior layers of the rectus sheath (2).

The sublay technique (7) and resection of the rectus diastasis and thinned linea alba followed by placement of sutures are open surgical procedures cited in the literature for repair of RAD with concomitant primary ventral hernia.

A third hybrid technique was pioneered by Reinpold (8) where the sublay technique is implemented *via* a minimal skin incision; this is known as the “mini-incision less open sublay” (MILOS).

Rectus abdominis diastasis is also often corrected in the course of abdominoplasty (9–20).

The traditional method of correcting RAD during abdominoplasty is by plicating one intact anterior rectus sheath against the opposite member (9, 12–19). This method usually creates significant tension as the anterior rectus fascia is advanced over the encased rectus muscles toward the midline. This technique may produce significant postoperative pain (12). To avoid these problems, Ramirez (12) has applied to his abdominoplasties a technique he has called “rectus abdominis myofascial release” (12). The first step is to incise the anterior rectus sheath at the junction of the inner one-third with the middle third of the rectus muscle width (12). These incisions meet at the level of the xiphoid process superiorly and the subumbilical area inferiorly (12). The rectus myofascial release allows the centrifugal forces of the rectus muscles to push the muscles toward the midline, facilitating closure with decreased tension (12).

The open rectus abdominis myofascial release method described by Ramirez (12) for RAD repair during abdominoplasty has been used by several working groups with good results for treatment of incisional hernias with reconstruction of the linea alba and mesh augmentation in open technique (21–26).

Below is now described the technique of endoscopic-assisted linea alba reconstruction plus mesh augmentation (ELAR plus) for treatment of RAD with concomitant primary ventral hernia, in addition to the early results. Like the MILOS technique described by Reinpold (8), this is a hybrid technique implemented *via* a small incision.

## Materials and Methods

### Operative Technique

**Figure 1:**
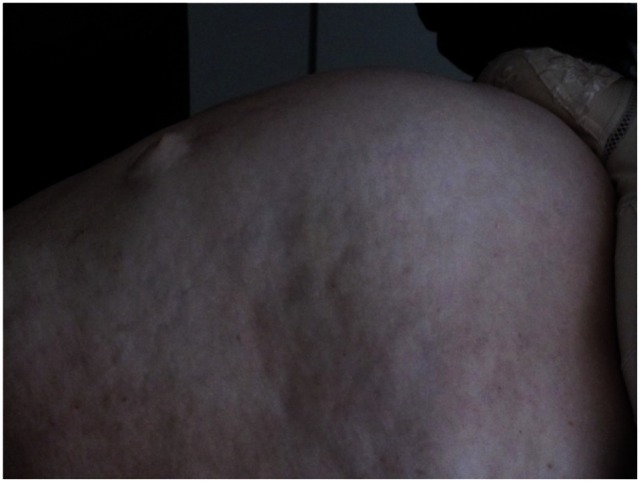
**Clinical findings of a patient with rectus abdominis diastasis, and umbilical and epigastric hernia when beginning to sit up**.

**Figure 2:**
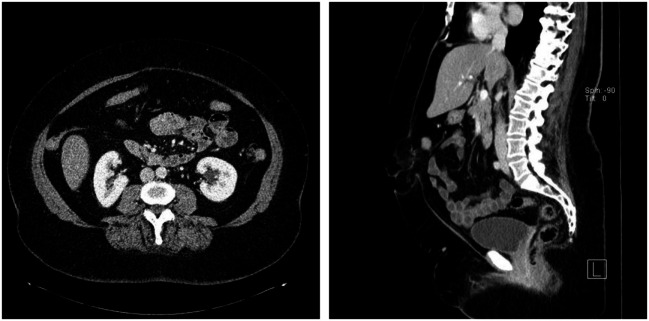
**CT scan of the patient with rectus abdominis diastasis, and umbilical and epigastric hernia**.

On raising their head and beginning to sit up from a supine position during clinical examination, all patients operated on in this technique exhibited marked bulging extending from the xiphoid process to the subumbilical area (Figure 1) Furthermore, all patients had a symptomatic umbilical hernia or epigastric hernia or both (Figure 2). Evidence of these findings was demonstrated on ultrasound, computed tomography (CT), and magnetic resonance imaging (MRI) (Figure 2).

In addition to repairing the symptomatic umbilical hernia and/or epigastric hernia with lasting closure of the hernia defect, surgery is also aimed at elimination of the actual cause, i.e., RAD. This is best done by reconstruction of a new linea alba from autologous tissue, followed by restoration of the rectus muscles to their normal anatomic position directly at the linea alba. Mesh augmentation is used to further stabilize the reconstructed abdominal wall. In order to be able to conduct this operation *via* a relatively small access route, a hybrid technique involving video-endoscopic equipment is used.

**Figure 3:**
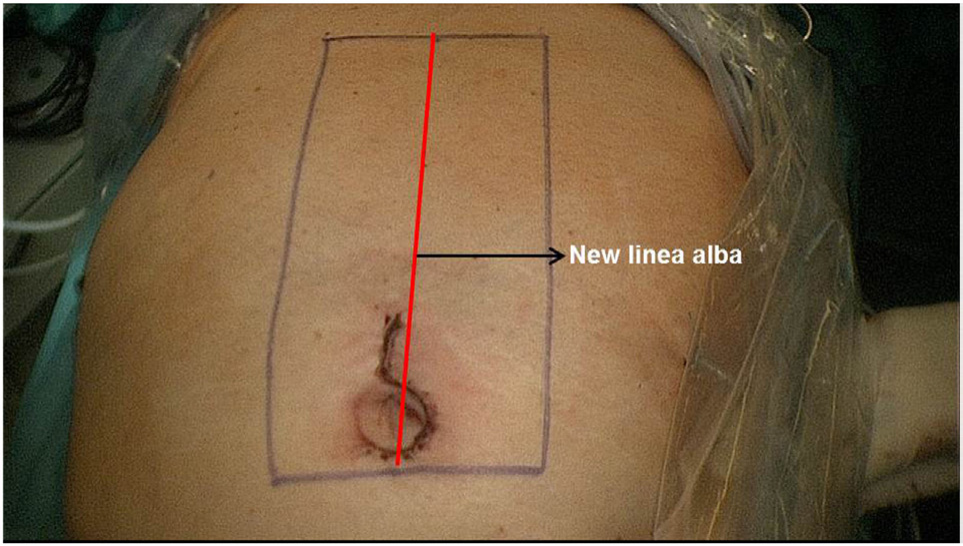
**Extent of incision and relation of mesh placement to linea alba reconstruction**.

The skin incision is made on the left side, encircling the umbilicus like a half-loop and extending 2–3 cm upwards (Figure 3). This is followed by stepwise diathermy dissection of the subcutaneous tissue, with detachment of the subcutaneous tissue from anterior rectus sheath on the left and right as well as below the umbilicus. Next, the umbilical hernia sac is opened, the hernia contents repositioned or resected and the umbilicus detached from the abdominal wall fascia. This is followed by further dissection beneath the abdominal skin/subcutaneous tissue and both anterior rectus sheaths using the video-endoscopic equipment. The operation can now be continued beneath the unopened abdominal skin as far as the xiphoid process by bilateral dissection of the anterior layers of the rectus sheaths. In those cases with an epigastric hernia, the hernia sac containing preperitoneal fatty tissue or parts of the greater omentum are detached from the subcutaneous fatty tissue and either repositioned or resected.

**Figure 4:**
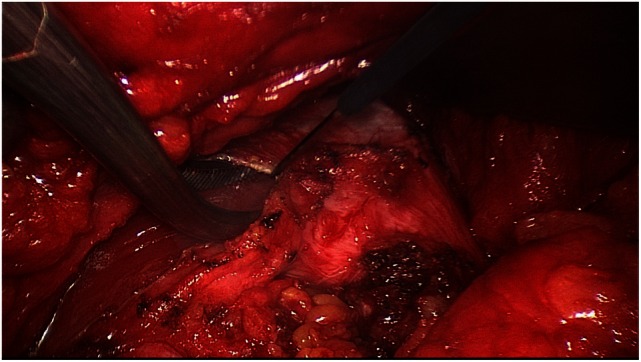
**Incision of right rectus sheath following dissection of the latter**.

**Figure 5:**
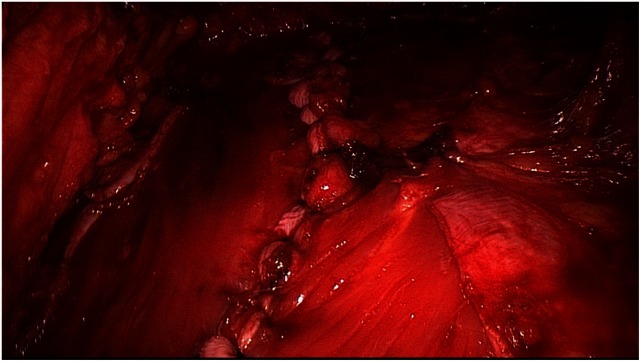
**New linea alba after suturing together the medial portions of the two rectus sheaths at the midline**. Both rectus abdominis muscles are repositioned beside the reconstructed linea alba.

**Figure 6:**
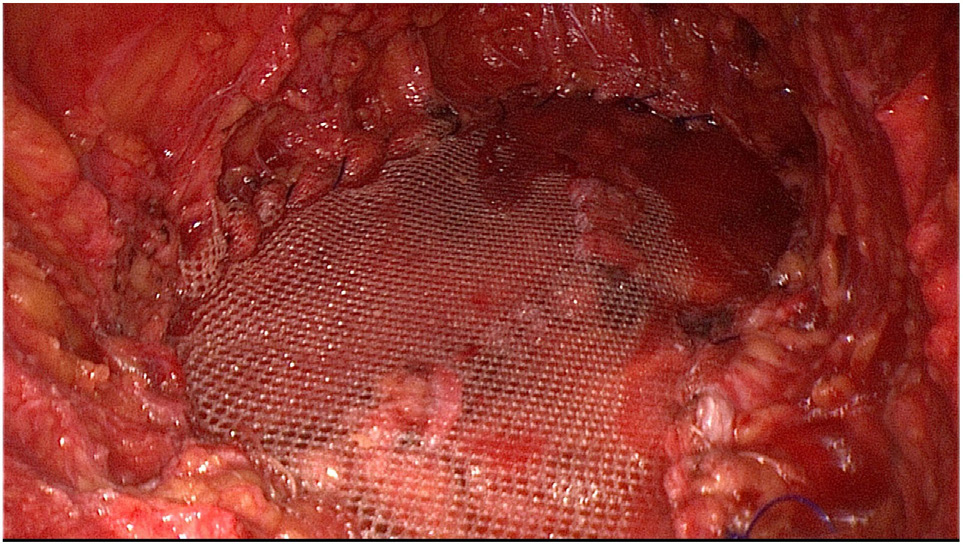
**Replacement of the medial segments of the rectus sheaths used to reconstruct the linea with a middle-weight, large-pore polypropylene mesh (TiMesh strong)**.

Next, both rectus sheaths are incised around 2–3 cm from the medial margin along their entire length from the xiphoid process to the subumbilical area and opened (Figure 4). That can be done with a scissors or the diathermy knife. After that, a new linea alba is reconstructed by suturing together the two medial parts of the right and left anterior rectus sheaths. Suturing is performed with non-absorbable suture material, assuring a stable new linea alba, with the rectus muscles restored to their normal anatomic position adjacent to the reconstructed linea alba (Figure 5). This suturing technique with reconstruction of the linea alba eliminates the RAD and closes the umbilical hernia and/or epigastric hernia defects. Using a continuous suturing technique, a mesh (TiMesh strong) for augmentation is then sutured to the incision margin of the right and left anterior rectus sheaths as replacement for the medial part of both anterior rectus sheaths (Figure 6). That completes reconstruction of the normal anatomy of the abdominal wall in this region. As a final step, a Redon drain is inserted between the mesh and subcutaneous tissue; the subcutaneous tissue is closed with single button sutures, finishing with an intracutaneous skin suture (Figure 3).

## Results

### 30-Day Postoperative Outcome

Between June 15, 2015 and January 31, 2016, 40 patients with RAD and symptomatic umbilical hernia and/or epigastric hernia underwent surgery. All patients were operated on by the senior author (Ferdinand Köckerling), who since January 1, 2000 had gained experience of implementing this reconstruction technique *via* a larger skin incision (25).

All patients were informed about the details of the procedure and gave their consent. The patients comprised 29 men and 11 women (Table 1). The mean age was 53.6 years. Patients had a mean BMI of 32.6. The mean ASA score was 1.9, and mean operating time was 120 min. The mean mesh (TiMesh strong) longitudinal length extension was 18.6 cm and transverse extension was 9.1 cm. Thirty-seven out of 40 patients did not have any postoperative complications (Table 2). One female patient had discrete partial umbilical necrosis, which dried out, healed, and was removed without redo surgery. It was possible to preserve the umbilicus and the wound healed without any sequelae. Besides, there was one case of postoperative seroma which did not require any further treatment. On 30-day follow-up, there was also one male patient with discrete impaired umbilical wound healing that also healed without further sequelae. Three out of 40 patients still complained about intermittent pain on exertion. Two out of 40 patients took painkillers when required (Table 3).

**Table 1:** **Patient characteristics, mean operating time, and mean mesh size**.

Sex	29 men/11 women	
Age	Mean: 53.6 years	25–76 years
BMI	Mean: 32.6	23–50
ASA	Mean: 1.9	1–3
OR duration	Mean: 120 min	67–179
Mesh length (TiMesh strong)	Mean: 18.6 cm	15–22
Mesh width (TiMesh strong)	Mean: 9.1 cm	5–13

**Table 2:** **Postoperative complication rate at the end of in-hospital treatment**.

None	*n* **=** 38/40	95%
Umbilical necrosis	*n* = 1/40	2.5%
Seroma	*n* = 1/40	2.5%

**Table 3:** **Results of 30-day follow-up visit**.

Discrete impaired umbilical wound healing	*n* **=** 2/40	5.0%
Intermittent pain on exertion	*n* = 3/40	7.5%
Painkillers still used when required	*n* = 2/40	5.0%

## Discussion

At present, there is no consensus among international hernia experts on the best surgical technique for repair of umbilical and epigastric hernias with concomitant RAD (2). Both open, e.g., sublay technique, and laparoscopic procedures are used (4). The open techniques requiring a larger incision are associated with more complications, such as impaired wound healing and wound infections, while the laparoscopic procedures are less efficient at eliminating rectus diastasis. Reinpold, Hamburg, has pioneered a third option with his MILOS technique (8), where using a hybrid technique, the sublay operation is performed *via* a very small incision (8). Inspired by his technique and experiences, we now set about modifying the technique we had practiced for more than 15 years for repair of ventral and incisional hernias. Details of this technique have been published in the literature under various names by Rehn (21), Chevrel (22), Abrahamson et al. (23), Flament et al (24), Schug-Pass et al. (25), and Joshi et al. (26). What these techniques have in common is that they all use a part of the anterior rectus sheath to reconstruct the new linea alba and thus close the hernia defects. Since implementation of this procedure involves a video-endoscopic technique, it constitutes a hybrid technique that is composed of an open and endoscopic approach. Accordingly, we have used the term “endoscopic-assisted linea alba reconstruction” to denote this technique. Since not all the techniques reported to date have used a mesh for augmentation (12, 23), we have expanded the term to include additionally “plus mesh augmentation.” The new technique which is performed *via* a markedly smaller access route can be standardized in a similar way to a procedure based on a larger incision. In none of the 40 operations conducted so far with this hybrid technique has it been necessary to switch to a larger incision, i.e., to the conventional procedure. The early postoperative 30-day follow-up results show a very low complication rate. No patient needed redo surgery because of postoperative complications. Only in two cases was discrete impaired umbilical wound healing observed, and these cases were successfully managed with conservative treatment while preserving the umbilicus. One case of discrete seroma was also easily managed. Only very few patients still experienced pain on exertion or required painkillers 4 weeks later. In all patients, both the umbilical and/or epigastric hernia and RAD were successfully eliminated and, accordingly, all patients were satisfied with the cosmetic results. Based on the longer term follow-up findings, the durability of these results will, of course, need to be demonstrated in the future. This is a clear limitation of this study. Our own experiences of carrying out this technique *via* a larger access route indicate that this surgical procedure which uses stable autologous tissue and is further underpinned by mesh augmentation produces good results with low recurrence rates (25). Likewise, in a recent publication, Joshi et al. (26) reported about a zero incidence of recurrence at a minimum of 12-month follow-up in 30 cases studied. As such, the basic principle enshrined in this technique holds out promising prospects for a good long-term outcome. The mini-incision procedure reduces the rate of wound complications, has fewer postoperative drawbacks for patients, and allows earlier resumption of everyday and working activities.

With the MILOS technique pioneered by Reinpold (8) and the ELAR plus procedure presented in this paper, there are now two new minimally invasive techniques available for treatment of ventral hernias and RAD. These are also suitable for repair of incisional hernias, since both the MILOS and ELAR plus surgical procedures can be used. Both techniques aim to reduce the drawbacks associated with the conventional open and endoscopic procedures used to repair ventral and incisional hernias. As such, they represent an alternative technique to the methods used to date. Already at this stage, there is reason to believe that the MILOS and ELAR plus techniques will in the future play a role in the “tailored approach” concept for treatment of ventral and incisional hernias.

## Ethics Statement

As the described operation technique was performed for many years *via* a larger incision in our hospital and results are reported in many publications in the literature, the approval by an ethics committee was not necessary.

## Author Contributions

FK, MB, and CR have treated the patients in their Hospital. The development of the study design and the follow-up of the patients have been also done by FK, MB, and CR. WR has first described abdominal wall hernia surgery *via* small incisions. FK, MB, CR, and WR are responsible for the content of the manuscript.

## Conflict of Interest Statement

The authors declare that the research was conducted in the absence of any commercial or financial relationships that could be construed as a potential conflict of interest.
